# Effects of water deficit at different stages on growth and ear quality of waxy maize

**DOI:** 10.3389/fpls.2023.1069551

**Published:** 2023-01-27

**Authors:** Chao Huang, Anzhen Qin, Yang Gao, Shoutian Ma, Zugui Liu, Ben Zhao, Dongfeng Ning, Kai Zhang, Wenjun Gong, Mengqiang Sun, Zhandong Liu

**Affiliations:** ^1^ Institute of Farmland Irrigation, Chinese Academy of Agriculture Sciences, Key Laboratory of Crop Water Use and Regulation, Ministry of Agriculture and Rural Affairs, Xinxiang, China; ^2^ College of Tobacco Science, Henan Agricultural University, Zhengzhou, China; ^3^ Guangli Irrigation Authority, Jiaozuo, China

**Keywords:** waxy maize, water deficit, leaf physiological characteristic, fresh ear yields, grain quality

## Abstract

**Introduction:**

Extreme weather has occurred more frequently in recent decades, which results in more frequent drought disasters in the maize growing season. Severe drought often decreases remarkably plant growth and yield of maize, and even reduces significantly the quality of maize production, especially for waxy maize.

**Results:**

To study the changes in plant growth, fresh ear yield, and fresh grain quality of waxy maize under water deficits occurring at different growth stages, and further strengthen the field water management of waxy maize, water deficit experiments were carried out under a rain shelter in 2019 and 2020. Water deficit treatments were imposed respectively at the V6–VT (D_V6–VT_), VT–R2 (D_VT–R2_), and R2–R3 (D_R2–R3_) stages of waxy maize, and treatment with non-water deficit in the whole growing season was taken as the control (CK). The lower limit of soil water content was 50% of field capacity for a water deficit period and 65% of field capacity for a non-water deficit period.

**Results:**

In this study, water deficits imposed at V6–VT and VT–R2 stages decreased plant growth rate and leaf gas exchange parameters, accelerated leaf senescence, and limited ear growth of waxy maize, which resulted in 11.6% and 23.1% decreases in grains per ear, 19.4% and 7.3% declines in 100-grain weight, 20.3% and 14.2% losses in fresh ear yield in 2019 and 2020 growing seasons, respectively, while water deficit at R2–R3 stage had no significant effect on ear traits and fresh ear yield, but the fresh ear yield with husk of DR2–R3 decreased by 9.1% (P<0.05). The obvious water deficit imposed at the V6–VT and VT–R2 stages also lowered grain quality. Water deficits at the V6–VT and VT–R2 stages led to accelerated maturity, resulting in increased total protein, starch, and lysine content in grains at the R3 stage and decreased soluble sugar content. Principal component analysis revealed that when water deficits occurred in the waxy maize growing season, they firstly altered maize physiological processes, then affected ear characteristics and yield, and finally resulted in significant grain quality changes. In conclusion, a water deficit during V6–VT and VT–R2 not only reduced fresh ear yield but also adversely affected grain quality. However, water deficit during R2–R3 had little effect on total protein, starch, and soluble sugar content,but increased obviously lysine content.

**Discussion:**

The above results suggested that avoiding serious water deficits at the V6–VT and VT–R2 stages of waxy maize while imposing a slight water deficit at the R2–R3 stage has not only little effects on fresh ear yield but also a remarkable improvement in grain quality.

## Introduction

Drought is one of the main disasters affecting agricultural production around the world. Climate change has led to the aggravation of drought in many regions and significantly increased the frequency of extreme drought (IPCC, 2014). Since 1950, the land area affected by drought in China’s agricultural production has shown a gradual upward trend, and the loss of food due to drought is about 25–30 × 10^6^ t, accounting for 60% of the total loss from natural disasters (Hao and Singh, 2015; Song et al., 2018).

Water deficit can lead to a large number of physiological stress reactions in plants, thus changing the physiological characteristics of plants, thereby affecting the growth of plants, and the yield and quality of final products (Wang and Frei, 2011). Under conditions of water deficit, plant cells will produce reactive oxygen species (ROS) due to oxidative damage and synthesize a large amount of malondialdehyde (MDA). Meanwhile, the enhanced activities of catalase (CAT), superoxide dismutase (SOD), and peroxidase (POD) prevent severe damage (Ye et al., 2020a). At the same time, soluble sugar, soluble protein, and proline content in plant cells will gradually increase to maintain normal cell osmotic pressure (Liu et al., 2015). Drought stress also significantly reduced the photosynthetic rate of maize leaves. On the one hand, stomatal opening would decrease under drought stress, leading to a decrease in CO_2_ supply and a decrease in the photosynthetic rate of maize leaves (Yao et al., 2012). On the other hand, peroxidation can reduce the activity of leaf photosynthetic enzymes (ribulose-1, 5-diphosphate carboxylase, and phosphoenolpyruvate carboxylase), resulting in a lower photosynthetic rate and final yield reduction of maize (Markelz et al., 2011; Ye et al., 2020a).

Water deficits can also affect the quality of crop products. Studies have shown that drought can reduce grain starch content and increase protein content in many crops (Wang and Frei, 2011; Thitisaksakul et al., 2012). Drought was shown to lower starch concentration in cassava tubers (Santisopasri et al., 2001), and water deficit during the flowering stage caused the process of starch accumulation in advance, and reduced the total starch accumulation (Yi et al., 2014). Some studies also showed that water deficit during the whole growth stage increased starch accumulation, starch accumulation rate, and the activities of key enzymes for starch synthesis (AGPase (glucose-1-ATP transferase), SS (starch synthase), and SBE (1,4-glucan branching enzyme)) at early filling stage in wheat, but decreased starch accumulation and amylose content at late filling stage (Dai et al., 2008; Singh et al., 2008). Studies also showed that drought stress increased total protein concentrations but decreased the contents of alcohol-soluble protein, glutenin, and oat protein contents in wheat grains (Begcy and Walia, 2015; Flagella et al., 2010; Ozturk and Aydin, 2004).

It was also reported that a water deficit reduced lysine content and increased protein content in maize grains and changed maize grain quality by increasing nitrogen, magnesium, zinc, and alcohol-soluble protein concentrations and reducing potassium and glutenin concentrations (Erbs et al., 2015). However, some studies suggested that drought at different growing stages had different effects on the grain quality of maize. Compared with normal irrigation, a drought at the whole growth stage was shown to decrease starch content by 3% and increase protein content in normal maize (Liu et al., 2013a). Sandhya et al. (2010) reported that drought stress at the seedling stage reduced the content of protein and starch in grains, while Ma et al. (2006) found that the content of protein and lysine in grains would be increased under moderate drought but decreased under excessive drought at the maize seedling stage. Drought imposed at the silking stage decreased starch content and increased protein content in maize grains (Wang and Frei, 2011; Thitisaksakul et al., 2012; Beckles and Thitisaksakul, 2014; Wang et al., 2021a). Drought stress at the flowering and post-anthesis stages both decreased grain protein content and fresh ear yield (decreased by 16.2%), but increased grain starch content in waxy maize (Zhao, 2017; Shi et al., 2018; Wang et al., 2021a). However, drought stress at the filling stage had no significant effects on the starch content but increased the protein content in the grains of fresh waxy maize. (Lu et al., 2015). In the process of grain formation, drought stress reduced the final starch content but increased the protein content (Wang et al., 2021b).

As a fresh-eating food, waxy maize places a high value on grain quality. Higher quality can bring better edible value and economic value (Wang et al., 2020). With the rapid improvement of citizen living standards, the planting area of fresh waxy maize increased significantly in the last decade in China. It can be expected that waxy maize will have a better market prospect and that the plantation area will continue to develop in the future. Although about 70% of the average annual rainfall of 582 mm occurred in the period of June to October in the Huang-Huai-Hai Plain (Si et al., 2020), most of the rainfall was given in several heavy rainstorms (Ma et al., 2016), which usually results in long periods without any effective rainfall and severe droughts during the maize growing season. The use of appropriate measurements and techniques is vital for high-yield, good-quality, and sustainable waxy maize production in the region. For the effects of water deficits on grain quality of waxy maize, most of the previous studies-imposed water deficits after flowering. They mainly explored the changes in grain quality of waxy maize at complete maturity under different water-deficit treatments. However, few studies have focused on the effects of water deficits occurring in the vegetative growth stage, especially on grain quality in the fresh stage. Therefore, the main objectives of this experiment were focused on: (1) clarifying the changes in plant growth, physiological characteristics, fresh ear yield, and fresh grain quality of waxy maize under water deficit at the jointing stage, flowering stage, and filling stage; and (2) revealing in detail the tolerance of waxy maize to water deficit at different growing stages for determining suitable water management during the waxy maize growing period. This research contributes to the rapid development of waxy maize production in the Huang-Huai-Hai Plain.

## Materials and methods

### Site description

**Figure 1:**
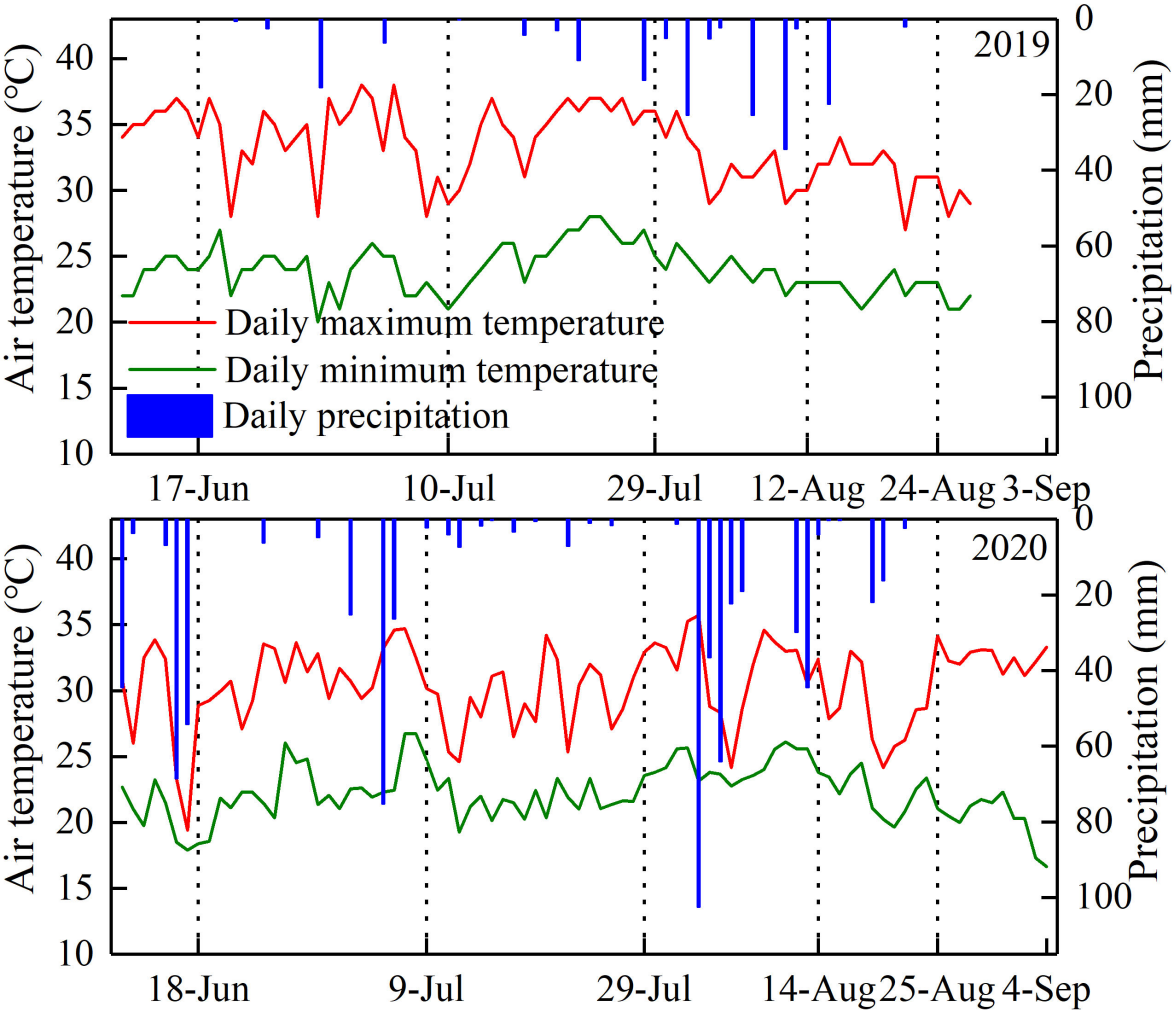
Daily air temperature and precipitation during the 2019 and 2020 growing seasons.

The experiment was carried out in lysimeters under a large-scale rain shelter at the Xinxiang Comprehensive Experimental Station of the Chinese Academy of Agricultural Sciences located in Qiliying Town, Xinxiang, Henan, China (35°18′N, 113°54′E, 75 m a.s.l.) with temperate monsoon weather in the 2019 and 2020 maize growing seasons. All lysimeters are non-weighing with well-equipped irrigation and drainage systems. The dimensions of each lysimeter were 2.0 m wide × 3.33 m length × 2.0 m in depth. The top side of the steel outer frame of the lysimeter is 10 cm higher than the soil surface in the lysimeter to prevent runoff during rain or irrigation events. A total of 24 lysimeters were arranged in two rows under a rain shelter. There was a 2 m space between the rows and 20 cm between lysimeters in the same row. The physical and chemical properties of the top 40 cm of the soil layer are shown in **Table 1**. A mobile rain shelter was installed above the two rows of lysimeters and closed before a rainfall and opened after the rainfall. This was done to avoid the severe effects of natural rainfall on the experiment of signed water deficit at different stages in maize growing seasons. An automatic weather station (YM-HJ03, Handan Chuangmeng Electronic Technology Co., Ltd., Hebei, China) was set on the edge of the lysimeter area. The average daily temperature and accumulated precipitation during the whole growing season of waxy maize in the two experiment seasons are shown in **Figure 1**.

**Table 1:** Basic parameters of topsoil of 0–40 cm in lysimeters.

Location	Soil texture	pH	Soil bulk density (g cm^−3^)	Soil field capacity (cm^3^ cm^−3^)	Organic matter (g kg^−1^)	Total nitrogen (g kg^−1^)	Available potassium (mg kg^−1^)	Total phosphorus (g kg^−1^)	Available phosphorus (g kg^−1^)	${\text{NO}}_{3}^{-}$ -N (mg kg^−1^)	${\text{NH}}_{4}^{+}$ -N (mg kg^−1^)
Xinxiang	Silt loam soil	8.8	1.51	0.31	10.72	0.73	138.96	0.94	72.00	18.34	2.54

Soil pH was determined in 1:5, soil to CO_2_-free water suspension by pH meter (120P-02A, Thermo Fisher Scientific); soil bulk density was measured by ring knife method; soil field capacity was measured by infiltration method; organic carbon was determined by potassium dichromate volumetric method; total nitrogen was determined by microcalorimetric method; exchangeable potassium was determined by flame photometric method; total phosphorus was determined by perchloric acid–sulfuric acid method; available phosphorus was determined by sodium hydrogen carbonate solution-Mo-Sb anti spectrophotometric method; ${\text{NO}}_{3}^{-}$ -N was determined by immerse-UV spectrophotometry method; ${\text{NH}}_{4}^{+}$ -N was determined by indophenol blue colorimetry.

### Experimental design

The experimental waxy maize variety was “Shenkenuo 1,” bred by the Shanghai Academy of Agricultural Sciences. The variety is a multi-resistant waxy maize variety with high taste quality and great planting promotion value (Wang et al., 2014). A total of 40 plants of waxy maize were maintained in each lysimeter, with row spacing of 60 cm and plant spacing of 30 cm. The experiment arranged only one factor (water deficit stage) with four treatment levels, i.e., water deficit occurred at V6–VT, VT–R2, and R2–R3 stages, and no water deficit in the whole waxy maize growing season (as CK), respectively. Following the soil water content arrangement shown in Xiao et al. (2011), no irrigation was carried out when the soil water contents were higher than 50% of field capacity during the process of water deficit treatment at the V6–VT, VT–R2, and R2–R3 stages of waxy maize, and irrigation was performed when the soil water content was reduced to or less than 50% of field capacity, or at the end of a growing stage with water deficit treatment. During the periods without arranging water deficit treatment, the soil water contents were maintained at more than 65% of field capacity, so the soil water contents in CK treatment were maintained at higher than 65% of field capacity in whole growing seasons. All four treatments were replicated three times. The planned soil water lower limits for all four treatments are shown in **Table 2**. The beginning date of each growth stage is shown in **Table 3**.

**Table 2:** Designed low limit of soil water content for different treatments at different waxy maize growing stages.

Water deficit treatment	Lower limit of soil moisture content			
	V1–V6 stage	V6–VT stage	VT–R2 stage	R2–R3 stage
CK	65	65	65	65
D_V6–VT_	65	50	65	65
D_VT–R1_	65	65	50	65
D_R1–R3_	65	65	65	50

The values in the table are the lower limit controlled of soil water content, and presented as percentage of field capacity. V1, first leaf; V6, sixth leaf; VT, tasseling; R2, blister stage; R3, milk stage.

**Table 3:** Beginning date of each growth stage of waxy maize in two growing seasons.

Year	Sowing	Second leaf	Sixth leaf	Tasseling	Blister stage	Milk stage	Harvest
2019	10.6.2019	17.6.2019	10.7.2019	29.7.2019	12.8.2019	24.8.2019	27.8.2019
2020	11.6.2020	18.6.2020	9.7.2020	29.7.2020	14.8.2020	25.8.2020	4.9.2020

### Measurement set-up

#### Measurements of soil water content

Soil water content (SWS, cm^−3^ cm^−3^) in the 0–100 cm soil layer was measured in real time with Insentek sensors (Oriental Zhigan Technology Ltd., Zhejiang, China) with a 10 cm increment. The sensor parameters were shown in Qin et al. (2019).

#### Measurements of plant height and leaf area index

At the six-leaf stage of waxy maize, three representative waxy maize plants with similar growth status were selected and marked in each lysimeter. The plant height and the leaf length and largest leaf width of all leaves on the three marked plants were measured at the end of each water deficit period of V6–VT, VT–R2, and R2–R3 in 2019 and 2020, and the leaf area index of each lysimeter was calculated by using Eq. (1) (Huang et al., 2022).


(1) $$LAI=0.75\times \frac{\Sigma_{i=1}^{m}\Sigma_{j=1}^{n}(L_{ij}\times W_{ij})}{m}\times \frac{N}{S}$$

where *LAI* is the leaf area index (dimensionless), *L_ij_* is the leaf length (cm) of the *j*th leaf on *i*th plant, *W_ij_* is the largest width (cm) of the *j*th leaf on *i*th plant, *m* is the measured number of plants, *n* is the number of leaves per plant, *N* is the plant number on a lysimeter, and *S* is the soil surface area of a lysimeter (cm^2^).

#### Measurements of gas exchange

A Li-6400 portable photosynthesis analyzer (LI-COR, USA) was used to measure the gas exchange parameters (including net photosynthetic rate (*P_n_*), stomatal conductance (*G_s_*), transpiration rate (*T_r_*), and intercellular CO_2_ concentration (*C_i_*)) of the ear leaves on the marked plants at the end of each water deficit period of V6–VT, VT–R2, and R2–R3 in 2019 and 2020. Measurements were carried out between 9:00 and 11:00 a.m. on a sunny day. A SPAD-502 portable chlorophyll meter (Konica Minolta Holdings, Inc., Japan) was used to measure the *SPAD* value of the ear leaves on the marked plants at the end of the three water deficit periods (Huang et al., 2022). The leaf water use efficiency (*LWUE*) was calculated with Eq. (2) (Huang et al., 2022).


(2) $$LWUE(\mu mol\;mmol^{-1})=\frac{P_{n}(\text{μmol }{\text{m}}^{-2}\;{\text{s}}^{-1})}{T_{n}(\text{μmol }{\text{m}}^{-2}\;{\text{s}}^{-1})}$$

#### Determination of enzyme activities and osmotic adjustment substances in waxy maize leaves

Five ear leaves of waxy maize in each lysimeter were sampled at the R3 stage in 2019 for determining the soluble protein content (determined with the BCA protein method), soluble sugar content (with the anthrone colorimetry method), proline content (with the ninhydrin method), and malondialdehyde (MDA, determined with the thiobarbituric acid method) contents of ear leaves of waxy maize. The methods of determining the activities of antioxidant enzymes such as superoxide dismutase (SOD, NBT method), peroxidase (POD, guaiacol method), and catalase (CAT, ammonium molybdate method) also were consistent with those described by Huang et al. (2022).

#### Fresh ear grain yield and ear characters

Fresh ears with husks were taken on all lysimeters (the harvest date is shown in **Table 2**) at the end of the R3 stage in the 2019 and 2020 seasons. A total of 20 representative ears were sampled on each lysimeter to measure the yield of fresh ears with husks and the yield of fresh ears without husks. Ear characters such as ear length, ear diameter, bald tip length, grain rows per ear, grains per row, and grains per ear were also determined simultaneously by averaging the relevant values of the 20 sample ears. After threshing, three groups of 100 grains were randomly sampled to determine the 100-grain weight for each experimental lysimeter.

### Grain quality

The waxy maize grains were collected at late R3 stages for determining fresh grain quality using the method described by Huang et al. (2022) in the 2019 and 2020 seasons. The soluble sugar content (determined with the anthrone colorimetric method), starch content (with the anthrone-sulfuric acid method), total protein content (with the total nitrogen content method, total protein content = total nitrogen content × 6.25), and lysine content (with the ninhydrin chromogenic method) of waxy maize grains were measured for each sample. The contents of amylopectin, amylose, gliadin, gluten, albumin, and globulin were measured by the Sanshu Bio-Tech company in China. The amylose content of starch was determined using a colorimetric amylose content assay (Knutson and Grove, 1994). The amylose content was analyzed using the GPC-RI-MALLs system developed by Park et al. (2007). Glutenins and gliadins were extracted and quantitated subsequently from two biological replicates by reverse-phase ultra-performance liquid chromatography (RP-UPLC) according to a method described by Han et al. (2015), and the sample size was modified in minor ways according to the protein concentration of waxy maize grain. The albumin and globulin content was analyzed using an automatic microplate reader (Multiskan GO, Thermo, USA).

### Statistical analysis

The effects of water deficits imposed at different stages on the waxy maize growth index, grain yield, yield components, and grain quality were analyzed by analysis of variance using the General Linear Model procedure (GLM) in SPSS 19.0 (IBM Inc., Chicago, IL, USA). Duncan’s new multiple range difference method was used to test the significance of the difference at the *P<*0.05 level. Figures were drawn with Origin 2017 (OriginLab, USA). Principal component analysis was used to determine the comprehensive impact of the water deficit.

## Results

### Effects of water deficits on plant height and leaf area index

**Figure 2:**
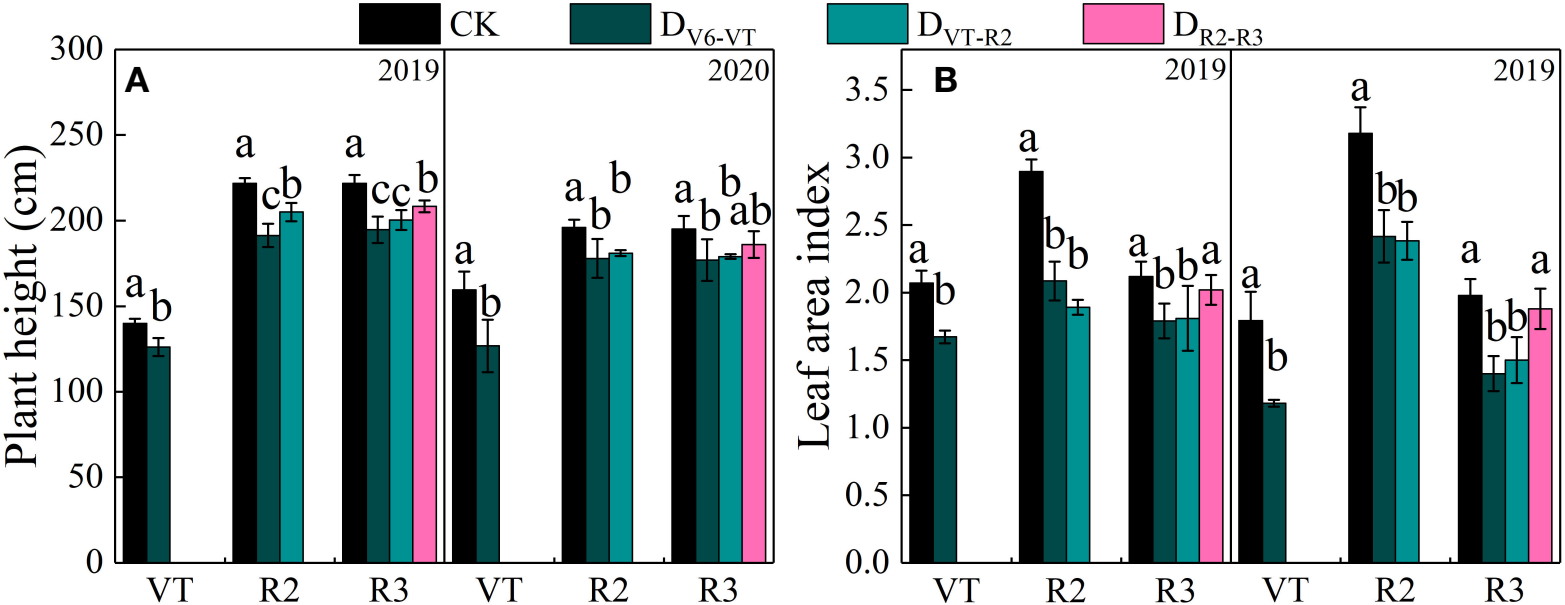
Plant heights and *LAI* of waxy maize under water deficits at different growing stages in the 2019 and 2020 seasons. Different lowercase letters during the same year indicated significant at 0.05 level. The X axes are years. CK, control; D_V6–VT_, water deficit from six leaf stage (V6) to tasseling stage (VT); D_VT–R2_, water deficit from tasseling stage to blister stage (R2); D_R2–R3_, water deficit from blister stage to milk stage (R3); VT, tasseling stage; R2, blister stage; R3, milk stage. **(A)** plant height of waxy maize; **(B)** leaf area index of waxy maize.

Plant height and leaf area index (*LAI*) of waxy maize varied significantly with growing stages and growing seasons (*P<*0.01; **Table 4**). The results of the two growing seasons showed that plant height and *LAI* decreased most significantly under the water deficit imposed at the V6–VT and VT–R2 stages (**Table 4**), and the change trends in the two growing seasons were basically the same (**Figure 2**). At the end of the water deficit period of V6–VT and VT–R2, all plant heights and *LAI* values of D_V6–VT_ and D_VT–R2_ were significantly lower than those of the CK treatment. Compared with CK, the plant height under D_V6–VT_ and D_VT–R2_ treatments at the R3 stage decreased by 10.7% and 9.0% (*P<*0.05), and the *LAI* decreased by 22.4% and 19.5% (*P<*0.05), respectively. The D_R2–R3_ treatment did not exhibit significant effects on plant height and *LAI*, but because of temperature, light, and other reasons factors, the plant height of waxy maize in 2019 was higher than in 2020. It was clearly indicated that a water deficit imposed at the V6–VT and VT–R2 stages may severely limit the plant growth and leaf development of waxy maize.

**Table 4:** ANOVA results of relevant waxy maize indices in the 2019 and 2020 growing seasons.

Effect		Plant height (cm)	Leaf area index	Ear length (cm)	Ear diameter (mm)	Bald tip length (cm)	Rows per ear	Grains per row	Grains per ear	100-grain weight (g)	Fresh ear yield with husk (t hm^−2^)	Fresh ear yield (t hm^−2^)	Total protein (mg g^−1^)	Grain soluble sugar (mg g^−1^)	Starch (mg g^−1^)	Lysine (mg g^−1^)
Stage	CK	213a	2.05a	17.35a	46.01a	1.16a	14.6ab	32.8a	505a	36.56a	15.24a	10.77a	80.52b	80.80a	77.55d	7.93b
	V6–VT	189c	1.60b	15.47c	46.33a	0.98a	14.0b	33.3a	453b	29.50c	12.51c	8.13d	87.37a	55.53c	161.66a	9.26a
	VT–R2	193c	1.65b	16.21b	44.25b	1.23a	12.7c	28.0b	402c	33.88b	13.75b	8.74c	86.26a	69.14b	145.32b	9.81a
	R2–R3	201b	1.95a	17.20a	45.38ab	1.02a	15.0a	32.2a	459b	35.93a	13.84b	10.11b	82.08b	77.41a	97.08c	9.26a
Year	2019	206a	1.93a	14.60a	47.04a	0.35a	15.0a	38.4a	590a	35.67a	15.54a	10.11a	88.53a	73.83a	107.32b	8.98b
	2020	184b	1.69b	13.64b	43.94b	1.85a	13.1b	24.8b	319b	32.27b	12.12b	8.76b	79.58b	67.62b	133.48a	9.14a
Stage		**	**	**	**	**	**	**	**	**	**	**	**	**	**	ns
Year		**	**	**	*	ns	**	**	**	**	**	**	**	**	**	*
Stage × Year		ns	ns	**	*	ns	ns	**	*	ns	Ns	ns	ns	**	**	ns

Same lowercase letters indicated no significance between different stages and years at a = 0.05. The mean is reported. *P<0.05, **P<0.01, ns P ≥0.05.

### Effects of water deficits on MDA, antioxidant enzymes, and osmotic adjustment substances in maize leaves

**Figure 3:**
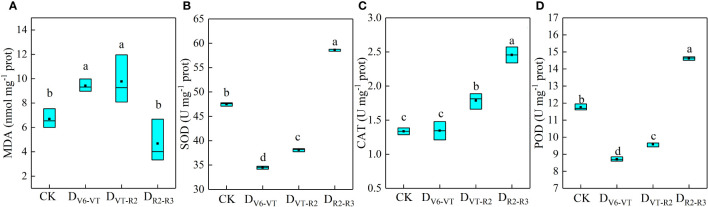
MDA contents and antioxidant enzyme activities of waxy maize leaves under water deficits at different growing stages in the 2019 season. Lowercase letters indicate the difference of different treatments at 0.05 level; MDA, malonaldehyde; SOD, superoxide dismutase; CAT, catalase; POD, peroxidase. the box from bottom to top indicated the lower quartile, median and upper quartile respectively, and the middle black box indicated the mean value. CK, control; D_V6–VT_, water deficit from six leaf stage (V6) to tasseling stage (VT); D_VT–R2_, water deficit from tasseling stage to blister stage (R2); D_R2–R3_, water deficit from blister stage to milk stage (R3). **(A)** MDA content of leaves; **(B)** SOD activities of leaves; **(C)** CAT activities of leaves; **(D)** POD activities of leaves.

MDA content and antioxidant enzyme activities in waxy maize leaves under different water-deficit treatments are shown in **Figure 3**. Compared with CK, the MDA content in D_V6–VT_ and D_VT–R2_ increased by 40.8% and 46.0%, respectively (*P<*0.05, **Figure 3A**), while the MDA content in D_R2–R3_ was significantly decreased by 30.1% (*P<*0.05). These results indicated that the recovery of plants in the D_V6–VT_ and D_VT–R2_ treatments was weak after re-watering. It may be the main reason that changes in CAT were insignificant under D_V6–VT_ treatment, and the activities of SOD and POD were significantly decreased by 26.8% and 25.8%, respectively, compared with CK (*P<*0.05). The oxidative damage to waxy maize plants still appeared obviously at the milk stage, even after a long-term release of the water deficit imposed at the jointing stage. D_VT–R2_ significantly increased CAT by 33.6% (*P<*0.05) but decreased significantly SOD and POD by 19.1% and 18.4% (*P<*0.05), which indicated that the oxidative damage caused by the water deficit at the VT–R2 stage can only be alleviated to a certain extent. D_R2–R3_ exhibited less oxidative damage and obvious increases in SOD, CAT, and POD by 24.5%, 83.8%, and 24.5%, respectively (*P<*0.05), which indicated that the damage caused by water deficit at the R2–R3 stage may have been almost completely alleviated at the milk stage.

**Figure 4:**
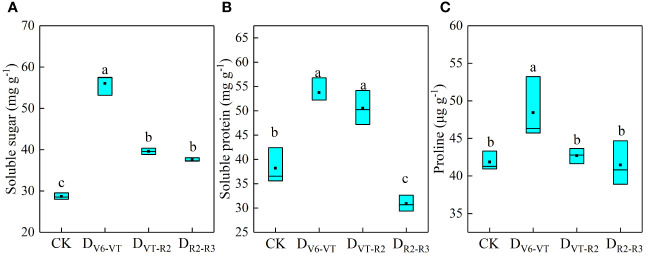
Contents of osmotic adjustment substances of waxy maize leaves under water deficits imposed at different growing stages in the 2019 season. Lowercase letters indicate the difference of different treatments at 0.05 level; the box from bottom to top indicated the lower quartile, median and upper quartile respectively, and the middle black box indicated the mean value. CK, control; D_V6–VT_, water deficit from six leaf stage (V6) to tasseling stage (VT); D_VT–R2_, water deficit from tasseling stage to blister stage (R2); D_R2–R3_, water deficit from blister stage to milk stage (R3). **(A)** soluble sugar contents of leaves; **(B)** soluble protein contents of leaves; **(C)** proline contents of leaves.

The soluble protein, soluble sugar, and free proline in waxy maize leaves at the R3 stage are shown in **Figure 4**. Results showed that the recovery of waxy maize plants after re-watering was poor under D_V6–VT_ and D_VT–R2_ treatments. Compared with CK, the soluble sugars and soluble proteins in the D_V6–VT_ treatment increased by 95.4% and 38.1% (*P<*0.05) and by 42.0% and 26.4% (*P<*0.05) in the D_VT–R2_ treatment, respectively. These increases were beneficial to maintaining cell water potential under water deficits, reducing leaf water loss, and improving leaf water use efficiency. The D_V6–VT_ treatment had the highest levels of soluble sugar, soluble protein, and proline. Meanwhile, the soluble sugar content in the D_R2–R3_ treatment increased by 30.9% (*P<*0.05), but the soluble protein decreased by 19.1% compared with the CK treatment. It was further indicated that a water deficit in the V6–VT stage had the greatest impact on the relevant index in waxy maize leaves and the smallest effects from a water deficit in the R2–R3 stage.

### Effects of water deficits on photosynthetic characteristics

**Figure 5:**
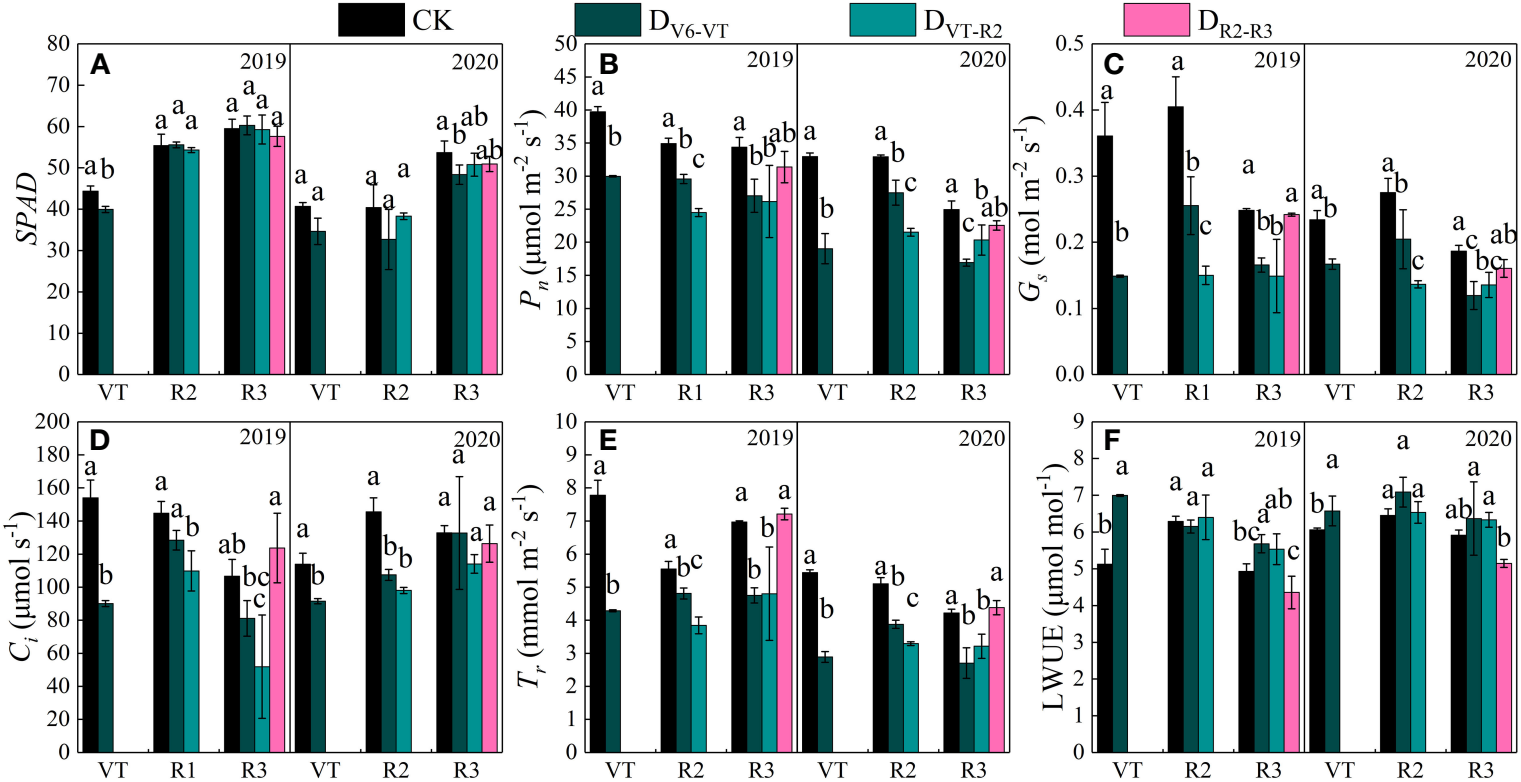
Photosynthetic characteristics of waxy maize leaves under water deficits at different growing stages in the 2019 and 2020 seasons. Different lowercase letters during the same stage indicated significant at 0.05 level. *SPAD*, leaf chlorophyll content index; *P_n_*, net photosynthetic rate; *G_s_*, stomatal conductance; *C_i_*, intercellular CO_2_ concentration; *T_r_*, transpiration rate; LWUE, leaf water use efficiency. The X axes are years. CK, control; D_V6–VT_, water deficit from six leaf stage (V6) to tasseling stage (VT); D_VT–R2_, water deficit from tasseling stage to blister stage (R2); D_R2–R3_, water deficit from blister stage to milk stage (R3). **(A)** SAPD value of leaves; **(B)** net photosynthetic rate of leaves, *P_n_*; **(C)** stomatal conductivity of leaves, *G_s_*; **(D)** intercellular CO_2_ concentration of leaves, *C_i_*; **(E)** transpiration rate of leaves, *T_r_*; **(F)** leaf water use efficiency of leaves, LWUE.

Under water deficit, plants usually reduced water loss by closing partially stomates, while the photosynthetic recovery of waxy maize was different after release from the water deficit imposed at different growing stages (**Figure 5**). The changes in *SPAD* and photosynthetic characteristics in the two growing seasons were basically the same (**Figure 5**). After water deficit at the V6–VT stage, *SPAD* and gas exchange parameters of leaves decreased, but leaf water use efficiency (*LWUE*) increased. After a water deficit at the VT–R2 stage, *P_n_*, *G_s_*, *C_i_*, and *T_r_* of leaves decreased significantly. Compared with CK, the D_V6–VT_ treatment reduced the *SPAD* of waxy maize leaves (**Figure 5A**), and the D_V6–VT_ and D_VT–R2_ treatments significantly decreased *P_n_*, *G_s_*, and *T_r_* (**Figures 5B, C, E**) and *C_i_* of waxy maize leaves at the R3 stage. Both D_V6–VT_ and D_VT–R2_ treatments increased *LWUE* significantly (**Figure 5F**). But no significant differences in *P_n_*, *C_i_*, *G_s_*, *T_r_*, and *LWUE* between D_R2–R3_ and CK were investigated. With the postponement of the water deficit stage, *P_n_*, *G_s_*, and *T_r_* showed an increasing trend, while *C_i_* showed a trend of decreasing at the beginning and then increasing later (**Figure 5D**), while *LWUE* showed a firmly decreasing trend (**Figure 5F**).

### Effects of water deficits on ear traits

**Figure 6:**
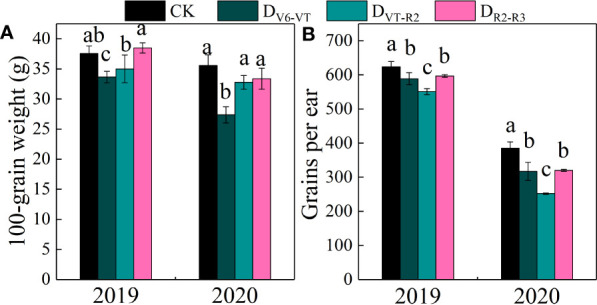
100-grain weight and grains per ear of waxy maize under water deficits at different growing stages in the 2019 and 2020 seasons. Different lowercase letters during the same stage indicated significant at 0.05 level. The X axes are years. CK, control; D_V6–VT_, water deficit from six leaf stage (V6) to tasseling stage (VT); D_VT–R2_, water deficit from tasseling stage to blister stage (R2); D_R2–R3_, water deficit from blister stage to milk stage (R3). **(A)** 100-grain weight of fresh grains; **(B)** grains per ear of fresh ear.

The results of the ANOVA in two growing seasons showed that there were significant differences in grains per ear and 100-grain weight under different water deficits imposed at different growth stages of waxy maize (*P<*0.01; **Table 4**). The 100-grain weight of D_V6–VT_ was significantly lower than those of other treatments, decreasing by 19.4% compared with CK (*P<*0.05; **Figure 6A**). Meanwhile, the grain yield per ear of the D_VT–R2_ treatment was the lowest and was 23.1% lower than that of CK (*P<*0.05; **Figure 6B**).

**Figure 7:**
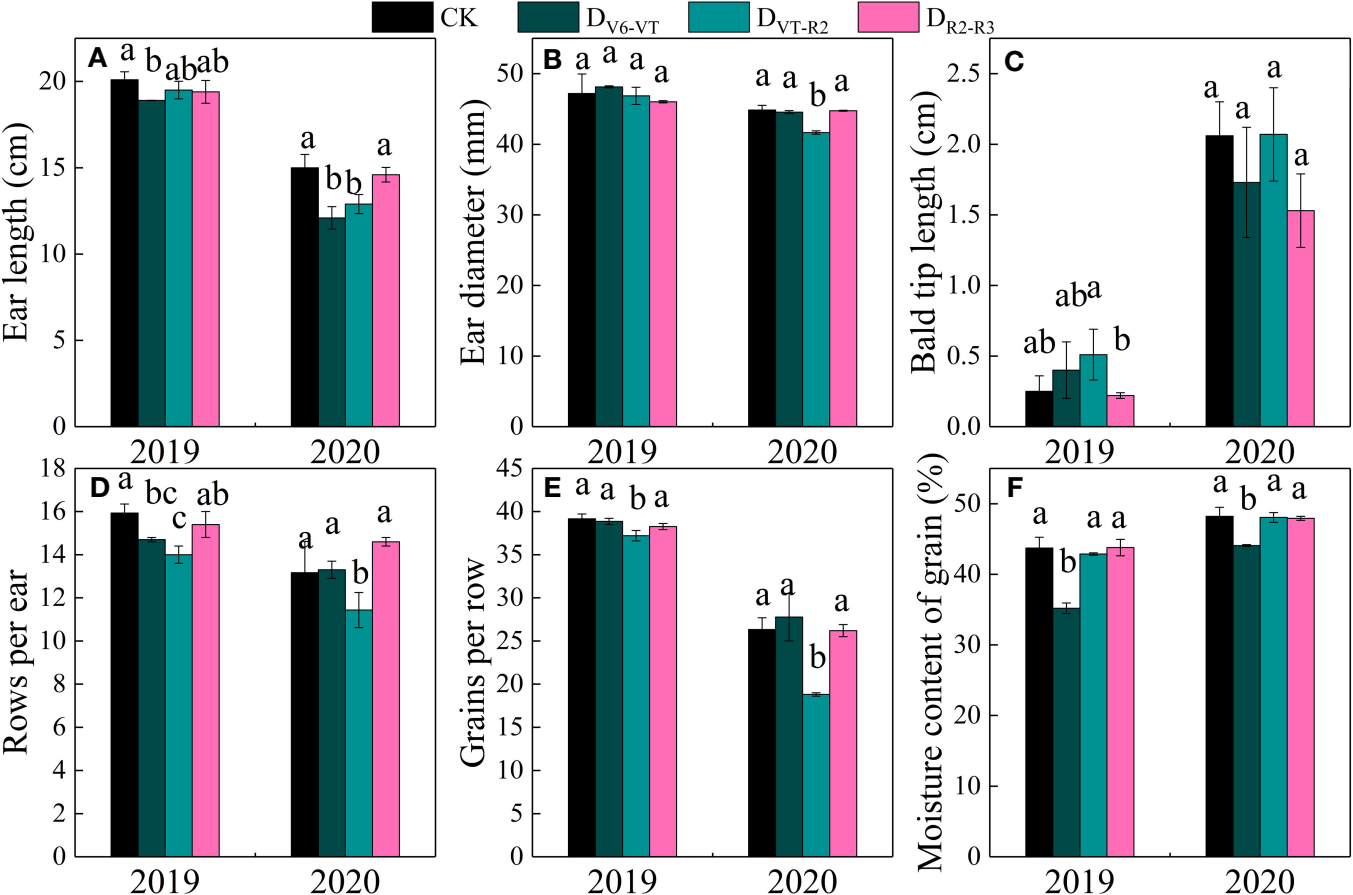
Ear traits of waxy maize under water deficits at different growing stages in the 2019 and 2020 seasons. Different lowercase letters during the same stage indicated significant at 0.05 level. The X axes are years. CK, control; D_V6–VT_, water deficit from six leaf stage (V6) to tasseling stage (VT); D_VT–R2_, water deficit from tasseling stage to blister stage (R2); D_R2–R3_, water deficit from blister stage to milk stage (R3). **(A)** ear length; **(B)** ear diameter; **(C)** bald tip length; **(D)** rows per ear; **(E)** grains per row; **(F)** moisture content of grains.

Ear length, ear diameter, bald tip length, grain rows per ear, and grains per row significantly varied with water deficit treatments (*P<*0.01; **Table 4**; **Figures 7A–E**). The results of multiple comparisons in two growing seasons showed that the ear length of D_V6–VT_, compared with CK, decreased the most obviously, followed by D_VT–R2_. Meanwhile, the ear diameter under the D_VT–R2_ treatment decreased the most significantly, and grain rows per ear and grains per row of D_VT–R2_ decreased significantly due to the decrease in ear length and ear diameter (**Table 4**). Compared with CK, the ear length of D_V6–VT_ and D_VT–R2_ decreased by 12.7% and 8.5%, respectively (*P<*0.05; **Figure 7A**), while the ear diameter, grain rows per ear, and grains per row of D_VT–R2_ decreased by 3.2%, 12.6%, and 16.8%, respectively (*P<*0.05; **Figures 7B, D, E**). It was the decrease in grain rows per ear and grains per row that resulted in the decrease in grains per ear. Due to the stage differences in imposed water deficit stages, the maturity dates of ears were obviously different, which resulted in different grain moisture contents at harvest. The grain moisture content of D_V6–VT_ treatment was significantly lower than that of CK, while no remarkable differences were investigated between the grain moisture contents of D_VT–R2_ or D_R2–R3_ treatments and that of CK at the end of R3 stage (**Figure 7F**).

### Effects of water deficits on fresh ear yield

**Figure 8:**
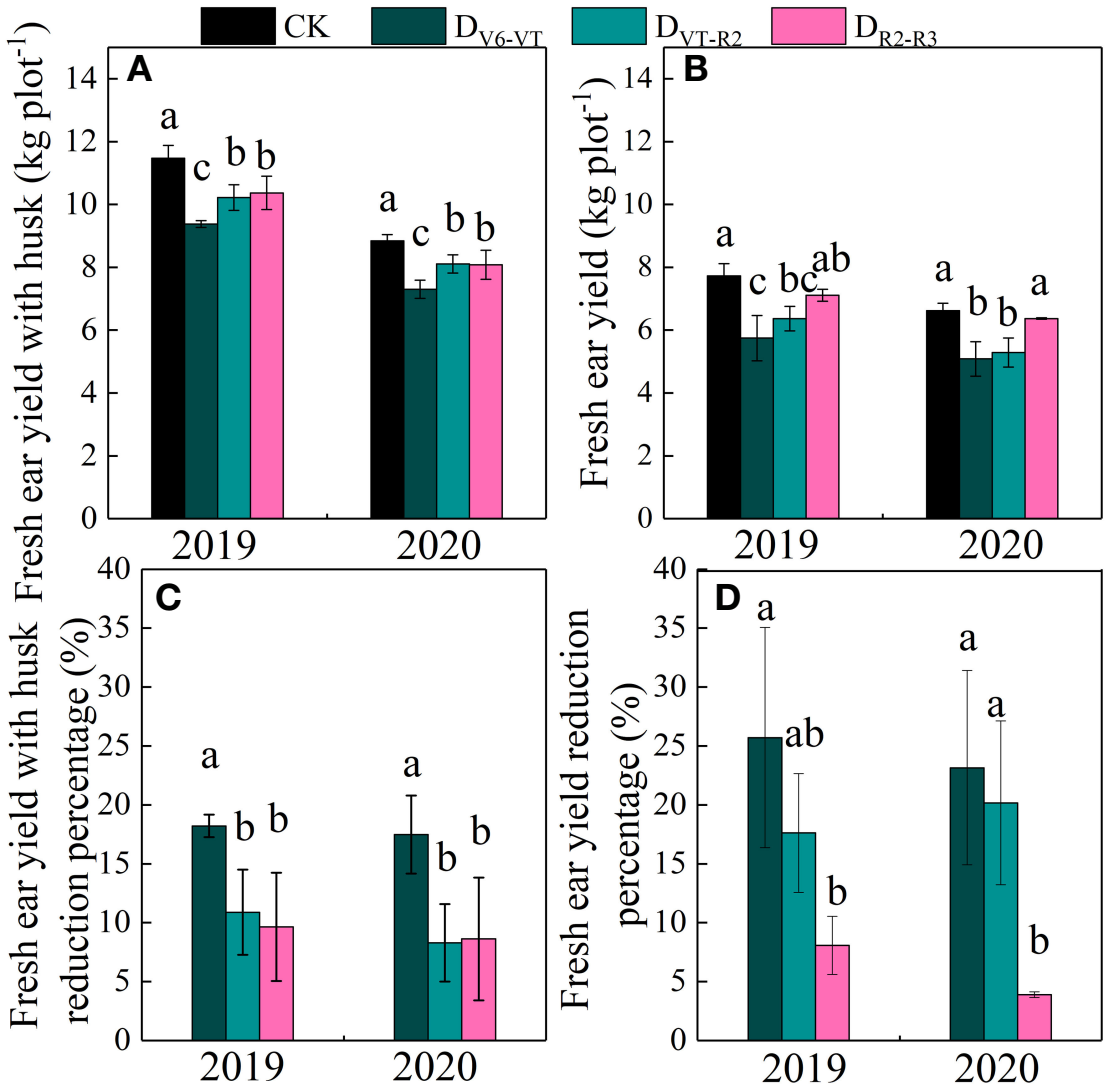
Ear yields of waxy maize under water deficits at different growing stages in the 2019 and 2020 seasons. Different lowercase letters during the same stage indicated significant at 0.05 level. The X axes are years. CK, control; D_V6–VT_, water deficit from six leaf stage (V6) to tasseling stage (VT); D_VT–R2_, water deficit from tasseling stage to blister stage (R2); D_R2–R3_, water deficit from blister stage to milk stage (R3). **(A)** fresh ear yield with husk; **(B)** fresh ear yield; **(C)** fresh ear yield with husk reduction percentage; **(D)** fresh ear yield reduction percentage.

Fresh ear yield with husks (HFY) and fresh ear yield (FY) were affected very significantly by the water deficit stage and growing seasons (*P<*0.01; **Table 4**). The ear yield of waxy maize varied under different water deficit treatments. Water deficit at the V6–VT stage showed the greatest effect on the ear yield, followed by those at the VT–R2 stage, and the least effect under water deficit at the R2–R3 stage (**Figure 8**). Compared with those under CK, mainly due to the varying grain number per ear and 100-grain weight under water deficit at different stages, the HFY and FY decreased by 22.0% and 20.3% under D_V6–VT_ (*P<*0.05), by 14.3% and 14.2% under D_VT–R2_ (*P<*0.05), and both less than 10.0% under D_R2–R3_, respectively.

### Effects of water deficits on grain quality

**Figure 9:**
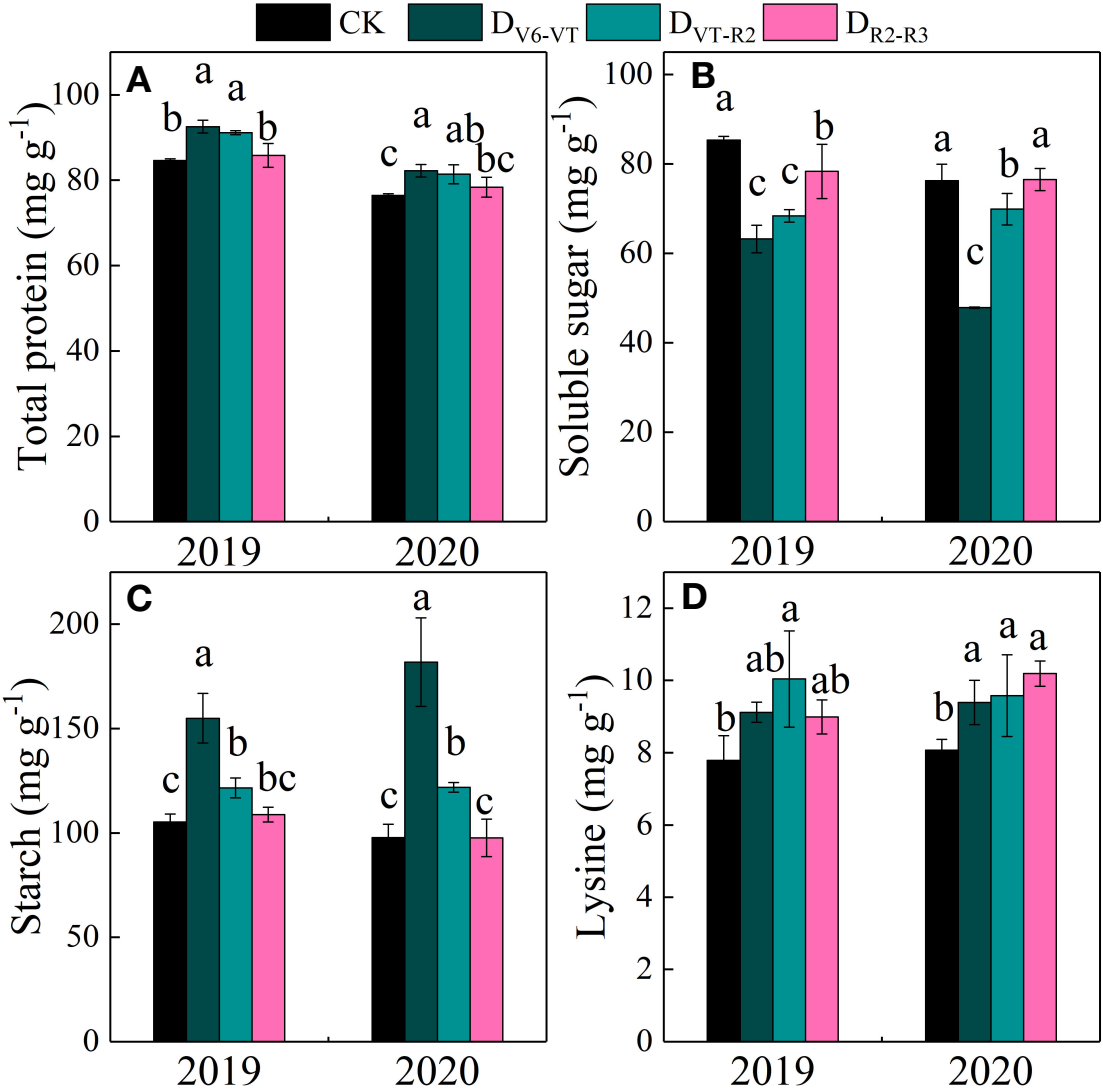
Grain quality traits of waxy maize under water deficits at different growing stages in the 2019 and 2020 seasons. Different lowercase letters during the same stage indicated significant at 0.05 level. The X axes are years. CK, control; D_V6–VT_, water deficit from six leaf stage (V6) to tasseling stage (VT); D_VT–R2_, water deficit from tasseling stage to blister stage (R2); D_R2–R3_, water deficit from blister stage to milk stage (R3). **(A)** total protein of fresh grains; **(B)** soluble sugar of fresh grains; **(C)** starch of fresh grains; **(D)** Lysine of fresh grains.

Total protein, grain soluble sugar, and starch contents in waxy maize grains varied significantly with the different water deficit stage treatments and growing seasons (*P<*0.01; **Table 4**). Water deficit increased the contents of total protein, starch, and lysine in fresh waxy maize grains, but reduced the content of soluble sugar in fresh waxy maize grains. However, the effects of water deficits at different growing stages on grain quality were obviously different, and the changing trends in the two growing seasons were consistent (**Figure 9**). The soluble sugar content of the D_V6–VT_ treatment was the lowest due to a 31.6% decrease over that of CK (**Figure 9B**; *P<*0.05), followed by the D_VT–R2_ treatment with a decrease of 14.1% (*P<*0.05), while the decrease was 4.0% under the D_R2–R3_ treatment. The total protein content and starch content were the greatest under the D_V6–VT_ treatment (**Figures 9A, C**) with an increase of 8.5% and 66.5%, respectively (*P<*0.05) than those under CK, respectively (*P<*0.05). The following were those under the D_VT–R2_ treatment with an increase of 7.1% and 20.1% (*P<*0.05), and under the D_R2–R3_ treatment with an increase of 2.0% and 1.5% under the D_R2–R3_ treatment. Compared with the CK treatment, the D_V6–VT_, D_VT–R2_, and D_R2–R3_ treatments increased the lysine content in grains by 16.7%, 23.8%, and 16.7%, respectively.

**Figure 9** indicated that the water deficit at the V6–VT stage had the most significant effects on the total protein and starch content of waxy maize grains, so the changes in amylose, amylopectin, and component protein content of waxy maize grains under the D_V6–VT_ treatment were selected as representative to show the effects of water deficit on grain quality index (**Table 5**). Compared with CK, the amylose and amylopectin content under the D_V6–VT_ treatment increased by 15.9% and 92.5% (*P<*0.05), the contents of glutelin and globulin decreased by 9.1% and 56.9%, respectively (*P<*0.05), while the contents of alcohol-soluble protein and albumin increased by 24.0% and 59.9%, respectively (*P<*0.05).

**Table 5:** Effects of water deficit imposed at jointing stage on starch and protein contents of waxy maize grains in the 2019 and 2020 growing seasons.

Year	Treatment	Amylose (mg g^−1^)	Amylopectin (mg g^−1^)	Alcohol soluble protein (mg g^−1^)	Glutenin (mg g^−1^)	Albumin (mg g^−1^)	Globulin (mg g^−1^)
2019	CK	33.69 ± 0.47b	71.61 ± 3.85b	24.00 ± 0.17b	37.86 ± 0.65a	2.97 ± 0.11b	1.55 ± 0.05a
	D_V6–VT_	36.44 ± 0.39a	118.55 ± 11.87a	31.54 ± 0.57a	35.19 ± 0.87b	4.59 ± 0.12a	0.67 ± 0.06b
2020	CK	34.28 ± 0.40b	63.55 ± 6.34b	26.76 ± 1.34b	32.58 ± 0.71a	3.33 ± 0.26b	1.50 ± 0.03a
	D_V6–VT_	42.39 ± 3.83a	139.46 ± 21.23a	31.23 ± 0.81a	28.95 ± 1.68b	5.50 ± 0.56a	0.65 ± 0.01b

The lowercase letters in the same column are the differences at the 0.05 level in the same year; the same uppercase letters are different between the two regions at 0.05 level. CK, control; D_V6–VT_, water deficit from six leaf stage (V6) to tasseling stage (VT).

### Principal component analysis

**Figure 10:**
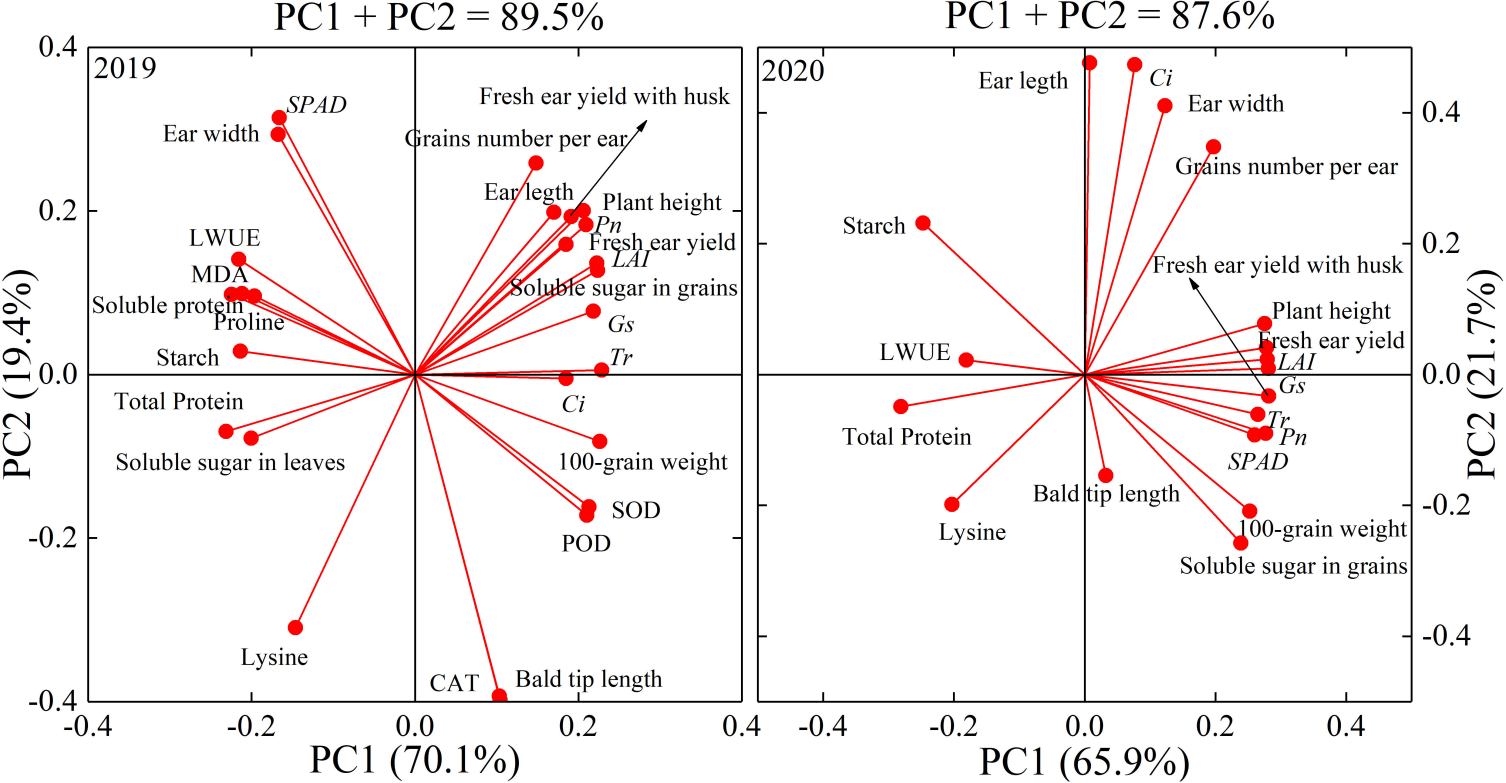
Loading diagram of principal component analysis in the 2019 and 2020 seasons. *LAI*, leaf area index; MDA, malonaldehyde; SOD, superoxide dismutase; CAT, catalase; POD, peroxidase; *SPAD*, leaf chlorophyll content index; *P_n_*, net photosynthetic rate; *G_s_*, stomatal conductance; *C_i_*, intercellular CO_2_ concentration; *T_r_*, transpiration rate; *LWUE*, leaf water use efficiency. PCn indicates the extracted principal component.

Principal component analysis (PCA) was performed on all measured indexes of waxy maize in the two growing seasons. Three principal components (PC1, PC2, and PC3) were extracted in 2019 and 2020 (λ >1), and the eigenvalues (λ) of principal component 1 (PC1) in 2019 and 2020 were 18.88 and 13.37, and explained 69.9% and 66.9% of the total variation, respectively. λ of PC2 in 2019 and 2020 were 5.19 and 4.22, which contributed 19.2% and 21.1% of the total variation, respectively. λ of PC3 in 2019 and 2020 were 2.93 and 2.41, which explained 10.9% and 12.0% of the total variation, respectively (**Table 6**). The largest loading variable in 2019 was CAT, followed by bald tip length, SPAD, lysine, and ear diameter. The largest loading variable in 2020 was *C_i_*, followed by ear length, ear diameter, grain number per ear, and fresh ear yield with husks. These results indicated that leaf physiology traits were most sensitive to water deficit, followed by ear traits and fresh ear yield in waxy maize (**Figure 10**).

**Table 6:** Eigenvalues and variances of principal component analysis in the 2019 and 2020 growing seasons.

Year	Principal Component Number	Eigenvalue (λ)	Total variation (%)	Cumulative (%)
2019	PC1	18.88	69.93	69.93
	PC2	5.19	19.20	89.14
	PC3	2.93	10.86	100.00
2020	PC1	13.37	66.86	66.86
	PC2	4.22	21.10	87.96
	PC3	2.41	12.04	100.00

## Discussion

### Effects of water deficits at different growing stages on leaf physiological characteristics of waxy maize

When plants are subjected to water stress, a large amount of reactive oxygen species will accumulate in the tissues, which will break the mechanism of reactive oxygen species production and scavenging, triggering the production of MDA by cell membrane peroxidation (Liu et al., 2015). Meanwhile, plant cells produce many kinds of antioxidant enzymes such as SOD, CAT, and POD to scavenge reactive radicals (Simova et al., 2008). Studies have shown that the accumulation of MDA varies with growing stages and that the longer a water deficit lasted, the more MDA was accumulated (Liu, 2013b). In our study, the MDA content in waxy maize leaves under the D_V6–VT_ and D_VT–R2_ treatments was significantly higher than that under the CK treatment at the R3 stage, and the MDA value under D_V6–VT_ was greater than that under D_VT–R2_. Our results are similar to the results of Li et al. (2017), which indicated that the magnitude of MDA accumulation at the jointing stage was greater than that at the filling stage. On the one hand, SOD, CAT, and POD activities in the cells increased to scavenge excessive reactive oxygen species (Liu et al., 2015). In this study, both SOD and POD activities were significantly lower in the D_V6–VT_ treatment compared with those in CK, mainly due to the inhibition of SOD and POD activities by excessive MDA (Liu, 2013b). But SOD, CAT, and POD activities in the D_R2–R3_ treatment increased significantly, which indicated that the plant functions recovered better after re-watering from the R2 to the R3 water deficit. Bu et al. (2009) also demonstrated an increase in protective enzyme activity after re-watering. Meanwhile, the increases in proline and total carbohydrate contents under water deficit are beneficial to protect maize plant tissues from oxidative damage (Bu et al., 2009; Anjum et al., 2016). Previous studies have shown that proline and MDA are in a reciprocal relationship, with proline accumulation helpful for reducing MDA damage to the plant, while soluble sugars and soluble proteins increase beneficial for maintaining cellular osmotic pressure (Liu et al., 2015; Li et al., 2017). In our study, the soluble sugar, soluble protein, and proline contents of waxy maize leaves under D_V6–VT_ were significantly higher than those under CK at the R3 stage, while these contents under both D_VT–R2_ and D_R2–R3_ were similar to those under the CK treatment. Even though the soluble sugar content increased after re-watering (Bu et al., 2009), the damage caused by the water deficit imposed at the V6–VT stage remained unrecovered. After re-watering, the recovery of antioxidant enzyme activity was very poor, photosynthesis was adversely affected, and an irreversible effect resulted in waxy maize plants grown under the D_V6–VT_ treatment in our study, mainly due to the long water deficit period (approximately 20 d, see **Table 3**) in the V6–VT stage.

Photosynthesis is the main physiological process driving plant growth and is highly sensitive to water deficits (Chaves et al., 2009). Water deficits reduce *P_n_*, *T_r_*, and *G_s_*, which in turn reduce maize plant biomass and grain yield (Ali and Ashraf, 2011; De Carvalho et al., 2011; Li et al., 2018). In our study, *P_n_* and *G_s_* decreased significantly under both the D_V6–VT_ and D_VT–R2_ treatments with nearly the same variation pattern, which was very similar to those results from Cai et al. (2017). *C_i_* was also significantly reduced in the D_VT–R2_ treatment, mainly due to stomatal limitation, but was not significantly affected in the D_V6–VT_ treatment. SPAD value decreased obviously under the D_V6–VT_ treatment, because reactive oxygen species generated by water deficit severely degraded severely chlorophyll pigments (Anjum et al., 2016) and significantly decreased chlorophyll content (Ye et al., 2020b). Water deficit also may reduce photosynthetic enzyme (PEPCase, RuBPase) activity (Ye et al., 2020a), and it caused a remarkable reduction in photosynthetic rate under the D_V6–VT_ treatment in this study. Reduced *G_s_* also limited transpiration and resulted in a significant decrease in *T_r_* under the D_V6–VT_ and D_VT–R2_ treatments. But *LWUE* increased when plants were exposed to a water deficit, due mainly to the obvious reduction in transpiration and water consumption (Yao et al., 2012).

### Effects of water deficits at different growing stages on yield and yield traits of waxy maize

The reduction of photosynthetic rate under water deficit may lead to a reduction of plant photoassimilate deposition and ultimately the loss of grain yield (Kimaro et al., 2015; Ye et al., 2020a; Ulfat et al., 2021). Previous studies have shown that a water deficit during grain filling reduced the grain fresh weight, water content, and grains per ear of waxy maize (Guo et al., 2022). In this study, fresh ear yield with husk and fresh ear yield were significantly reduced, and the grain number per ear and 100-grain weight also showed some reducing trends under the D_V6–VT_ and D_VT–R2_ treatments. The most obvious decrease in grain number per ear was investigated in the D_VT–R2_ treatment, while the most remarkable decrease in 100-grain weight occurred in the D_V6–VT_ treatment. Previous results already indicated that a water deficit imposed at the tasseling, flowering, and filling stages could reduce the grain number per ear and grain weight of waxy maize, resulting in yield loss (Sun, 2014). Similar to the results of Xiao et al. (2011), the yield loss under treatment with a water deficit that occurred at the jointing stage was less than that under a water deficit imposed at the filling stage. The yield loss trend under water deficit may be described as that the yield loss reduces gradually as the period of water shortage becomes later and later. The yield reduction was mainly caused by the decrease in grain number per ear and 100-grain weight (Wen, 2020). Yang et al. (2019) also showed that a water deficit imposed at the tasseling stage significantly reduced the grain number per ear and 100-grain weight of fresh waxy maize, mainly due to the decreases in pollen dispersal and grain filling rates. Water deficit not only affects the formation and vitality of pollen but also affects the differentiation of maize spikelets (Li et al., 2022), resulting in a decrease in the number of rows per ear, grain number per ear, and final yield reduction. This study showed that high accumulation of MDA in leaves resulted in quicker leaf senescence, and then decreases in *LAI*, photosynthesis rate, and dry matter accumulation under the D_V6–VT_ treatment. The greatest reductions in 100-grain weight and grain moisture content were investigated under D_V6–VT_ in this study, indicating that the D_V6–VT_ treatment caused the process of starch accumulation in advance and led to poor filling quality. The *P_n_* values in 2020 were generally lower than those in 2019, mainly due to less radiation and a lower temperature in 2020 (**Figure 5B**), which resulted in a lower 100-grain weight and less dry matter accumulation in 2020 (Gao et al., 2017). The worse radiation environment in the 2020 growing season also led to a decrease in pollen viability and a subsequent decline in grains per ear, which may be the main reason for the remarkable differences in fresh ear yields between the two growing seasons (Zhou et al., 2013).

### Effects of water deficits at different growing stages on grain quality of waxy maize

Grain quality was closely related to leaf physiology and yield. Studies have shown that an increase in wheat grain protein content is closely associated with a decrease in yield under water deficit (Ozturk and Aydin, 2004; Flagella et al., 2010). It is also indicated that the starch content of grain was also closely related to yield in barley (Worch et al., 2011). In this experiment with waxy maize, the D_V6–VT_ and D_VT–R2_ treatments increased the total protein contents while decreasing the soluble sugar contents. In addition, the starch and lysine contents were increased by the water deficit imposed at all growing stages. Several studies have shown that water deficit at the grain formation stage may reduce the starch content and increase the total protein content of waxy maize grain (Sun, 2014; Wang et al., 2021b). The increase in protein content was mainly due to the concentration effect of reduced biomass under water deficits (Rotundo and Westgate, 2009). Ma et al. (2006) also showed that the protein and lysine contents of maize grains increased under moderate water deficit conditions. The changes in protein and lysine contents in this study showed very similar trends to those in Ma et al. (2006). D_V6–VT_ resulted in increases in glutenin and albumin contents, but decreases in alcoholic-soluble protein and globulin contents in grains. For the grain quality of waxy maize, the effects of water deficit on grain starch content in this study were opposite to those shown in some previous studies (Sun, 2014; Wang et al., 2021b), maybe mainly because the previous results were investigated from matured grains, whereas fresh grains were used in this study. Debon et al. (1998) and Silva et al. (2001) found that the increase in the starch concentration of fresh fruit of potato was mainly due to the concentration effect caused by the decrease in water content in fresh fruit. Guo et al. (2022) and Shi et al. (2018) also showed an increase in starch content in fresh waxy maize grains under drought stress and indicated that acceleration of grain filling and decreases in grain water content under drought (**Figure 7F**) might be the main reasons for the starch content increase. Meanwhile, water deficit caused a significantly increase in abscisic acid (ABA) content in maize plant tissues, and then led to the activity increases of four key enzymes related closely to converting soluble sugar into starch (Zhang et al., 2012). Yi et al. (2014) indicated that a water deficit imposed at V6–VT advanced the starch accumulation process in sorghum, and Dai et al. (2008) also indicated that a water deficit increased starch accumulation in wheat at the early filling stage. In our study, both amylose and amylopectin increased under water deficit at the V6–VT period, and the percentage of amylopectin increase was much greater than that of amylose. Water deficit reduced the expression of starch branching enzymes SEBI and SBEIIb genes, which resulted in decreases in amylose and amylopectin content, and then the final total starch content (Wu et al., 2022). The results of PCA (**Figure 10**) showed that the antioxidant enzymes and gas exchange parameters in leaves were items affected early by water deficit, and ear growth, grain number per ear, 100-grain weight, the water content of fresh grains were affected lately, and fresh ear yield and grain quality as the finally affected items.

## Conclusion

The water deficit imposed at the V6–VT stage limits severely the growth of waxy maize plants in the vegetative stage, reduces the 100-grain weight of waxy maize, and results in a significant reduction in fresh ear yield and grain starch accumulation, mainly due to accelerated grain ripening. A water deficit imposed at the VT–R2 stage affects the flowering and pollination of waxy maize plants and results in a significant reduction in grain number per ear and the final ear yield. However, a moderate water deficit imposed at the R2–R3 stage had little effect on growth and development, fresh ear yield, total protein, soluble sugar, and starch content in fresh grains of waxy maize but had significant effects on increasing the lysine content in fresh grains. The results in this study also suggested that more in-depth and comprehensive studies on the effects of water deficit duration imposed at different growing stages should be performed, and suitable techniques for reducing loss of yield and quality caused by water deficit should be developed.

## Data availability statement

The raw data supporting the conclusions of this article will be made available by the authors, without undue reservation.

## Author contributions

CH: investigation, data curation, formal analysis, visualization, and writing—original draft preparation. AQ: conceptualization, methodology, data curation, formal analysis, writing, and manuscript reviews. YG and SM: methodology, writing—editing and funding acquisition. ZuL, BZ, and DN: provided guidance and manuscript reviews. WG and MS: investigation. KZ and ZiL: conceptualization, writing—editing, supervision, project administration, and funding acquisition. All authors contributed to the article and approved the submitted version.
